# Shoulder Pain Due to Humeral Metastasis: Unusual Presentation of Nasopharyngeal Carcinoma

**DOI:** 10.7759/cureus.96567

**Published:** 2025-11-11

**Authors:** Suvarna Dhake, Rushikesh Naik

**Affiliations:** 1 Radiology, Czech Rehabilitation Hospital, Al Ain, ARE; 2 Radiology, Burjeel Hospital, Abu Dhabi, ARE

**Keywords:** aggressive bone tumor, atypical presentation shoulder pain, humeral bone metastasis, nasopharyngeal carcinoma (npc), unusual metastasis

## Abstract

Nasopharyngeal carcinoma is the most common head and neck malignancy arising from the mucosal lining epithelium of the nasopharynx, the commonest presentation being a unilateral neck mass. While bone, liver and lung metastases are well-documented, the presentation with shoulder pain is unusual. We present an unusual case of a 16-year-old male with persistent shoulder pain not resolved with physiotherapy. A month later, a growing neck mass was reported. MRI shoulder revealed an aggressive bone lesion in the proximal humerus. MRI head and neck revealed a nasopharyngeal mass with right deep cervical lymphadenopathy. Biopsy of the humeral mass revealed undifferentiated nasopharyngeal cancer.

## Introduction

Nasopharyngeal carcinoma (NPC), a type of epithelial cancer originating from the nasopharyngeal mucosa, accounts for approximately 70% of primary malignancies of the nasopharynx with highly uneven geographic distribution [1]. It is strongly associated with the Epstein-Barr virus. NPC is quite common in the Mediterranean basin, especially so in North African countries [2].

NPC is commonly diagnosed between 40 and 60 years of age. It accounts for approximately 1%-3% of malignant tumors and 20%-50% of primary nasopharyngeal malignancies in pediatric age group. Two peaks of incidence have been observed in endemic countries, first one being 10-20 years and second peak between 40 and 60 years [3,4]. NPC has high frequency of distant metastasis; common sites being bones, lungs and liver [5].

The most common presentation of NPC is unilateral neck mass seen in approximately 80% of cases [6]. Metastatic deposits are extremely rare presenting symptoms seen in approximately 0.3% of cases [7]. The case we are presenting here highlights a rare presentation of NPC with shoulder pain due to an aggressive humeral mass that may masquerade as a primary bone tumor. The common and recognizable symptom of growing neck mass was not apparent at the initial visit. We present this case to bring into focus the unusual presenting symptom that may lead to misleading diagnosis and delay in definitive treatment with an aim to create awareness amongst the referring physicians and radiologists about this rare presentation.

## Case presentation

**Figure 1:**
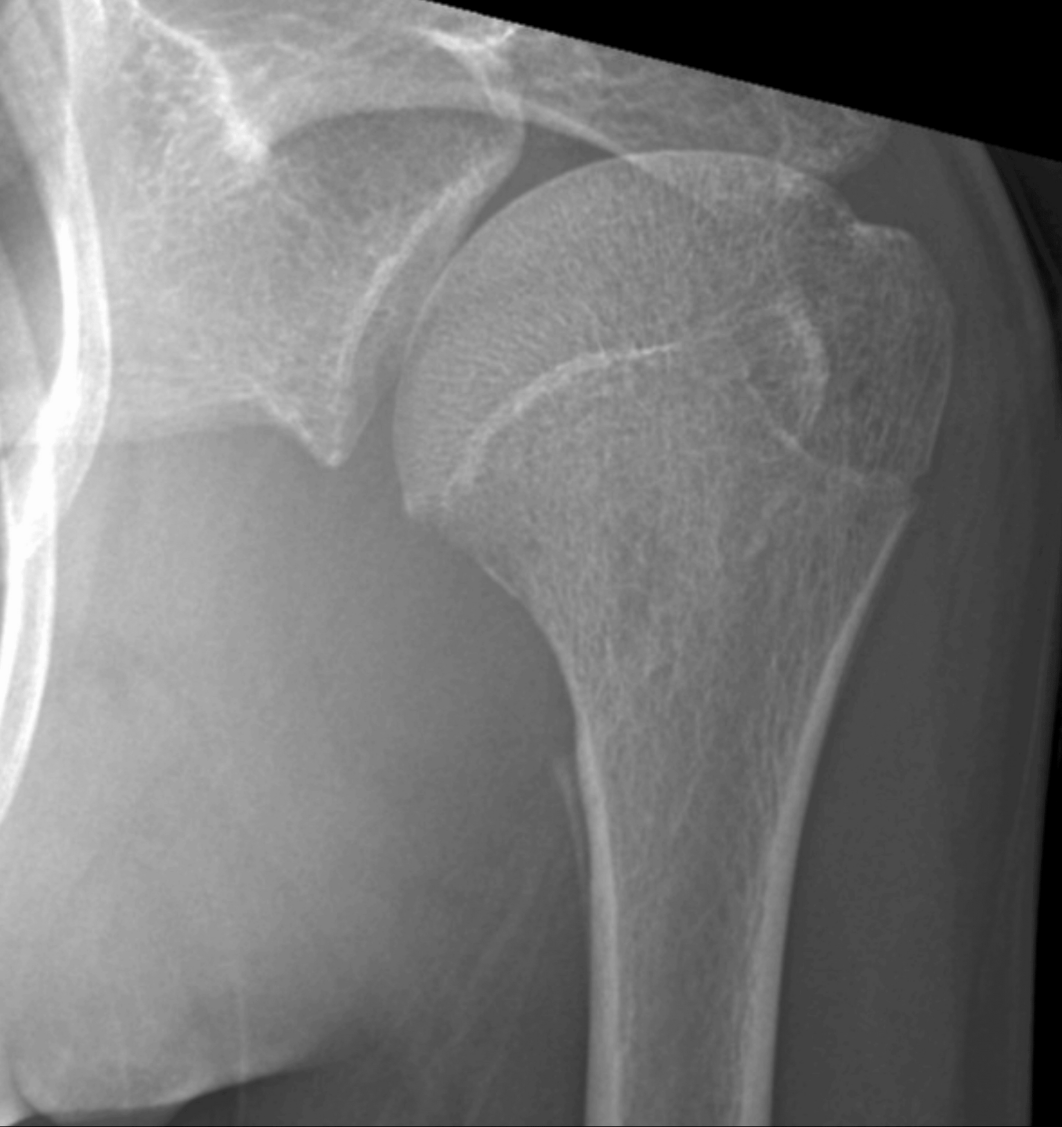
Frontal radiograph of the left shoulder Subtle periosteal reaction is seen along the medial aspect of proximal humerus. Also noted is a small area of ill-circumscribed erosion along the inferomedial aspect of humeral head.

A 16-year-old male presented with pain in the left shoulder after a fall in the washroom following a fainting episode in February 2025. The initial X-ray of the shoulder was unremarkable, and physiotherapy was advised. The X-ray was repeated in March 2025 due to persistent pain and restricted shoulder movement. X-ray of the shoulder revealed a subtle periosteal reaction along the medial aspect of the proximal humerus. A subtle area of erosion was seen along the inferomedial aspect of the humeral neck (Figure 1).

**Figure 2:**
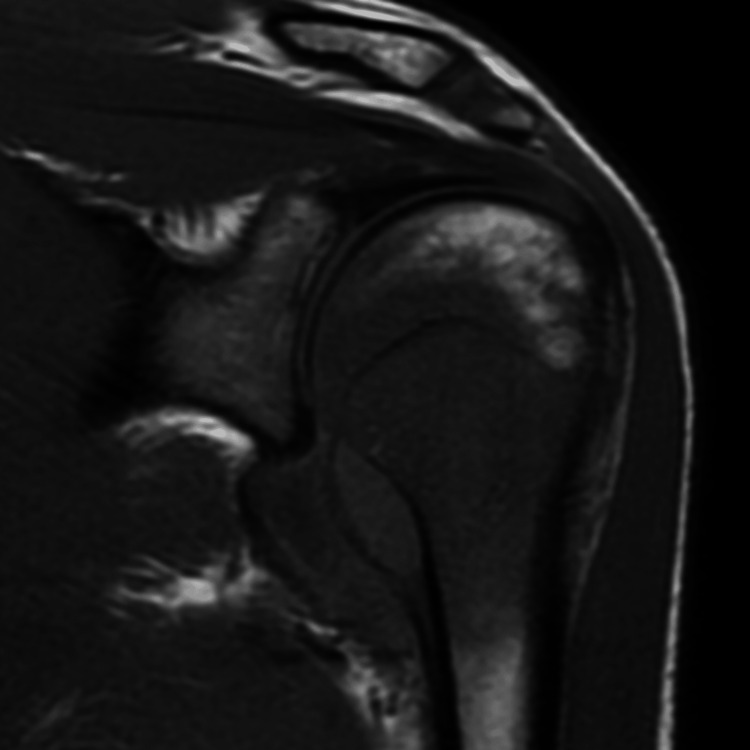
T1-weighted coronal image of the left shoulder A large mass is seen in the head and neck of the humerus with subperiosteal component. Also seen is elevated periosteum.

**Figure 3:**
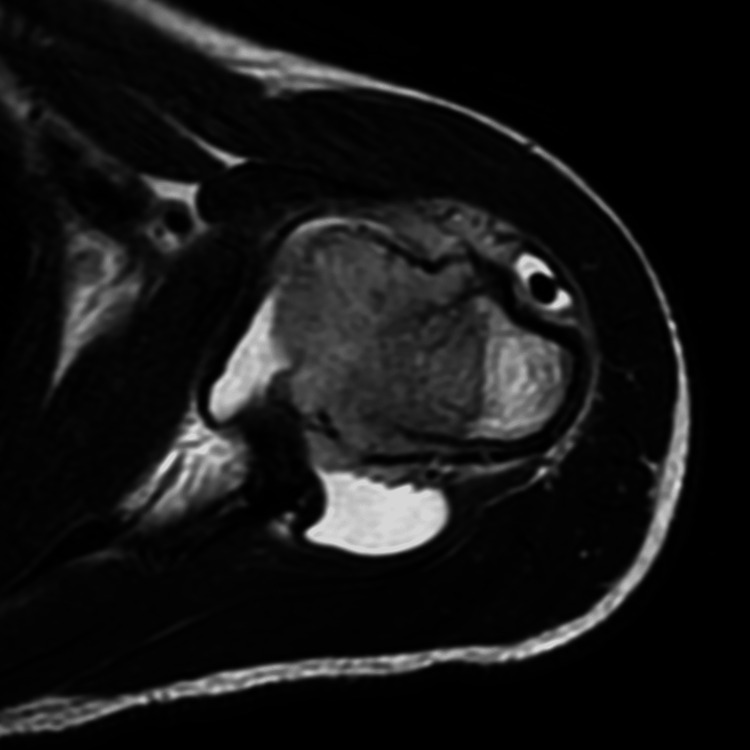
T2-weighted axial images at the level of humeral neck Extensive subperiosteal component of the mass is noted with breach of cortex as well as the overlying periosteum.

**Figure 4:**
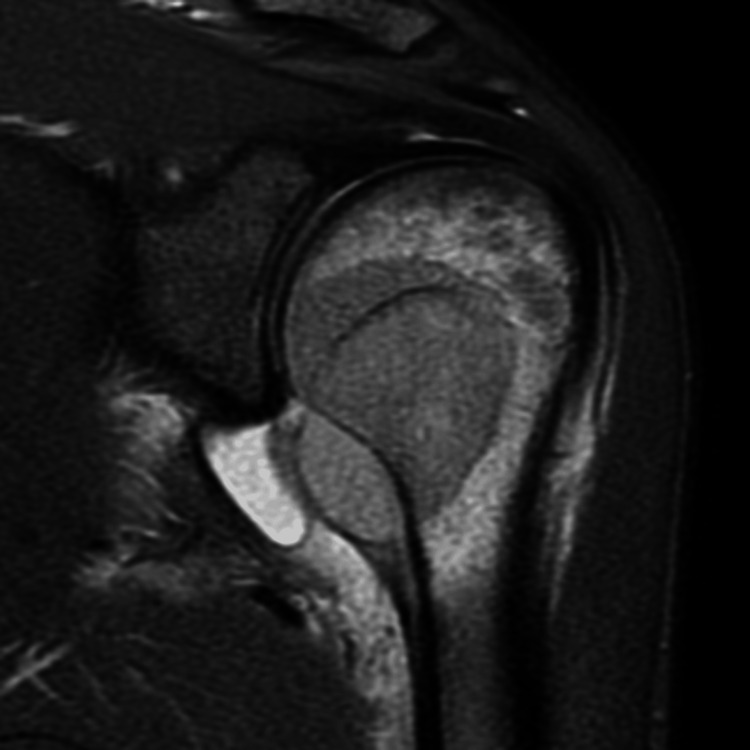
PD-weighted coronal image of the left shoulder The mass is demonstrated with marked adjacent marrow edema. PD: proton density.

**Figure 5:**
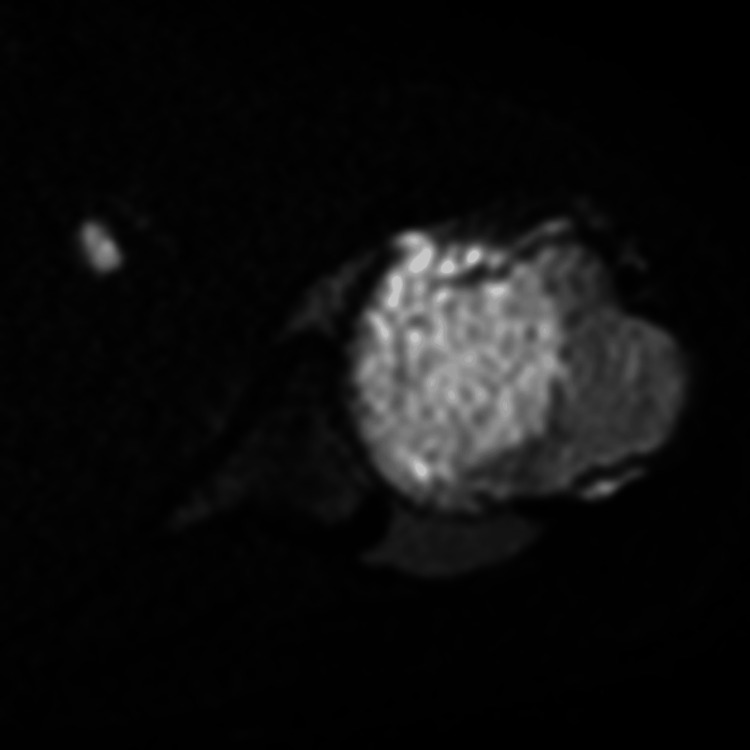
Diffusion-weighted imaging at the level of humeral neck The mass displays restricted diffusion. Also noted is restricted diffusion in the enlarged left axillary lymph node.

**Figure 6:**
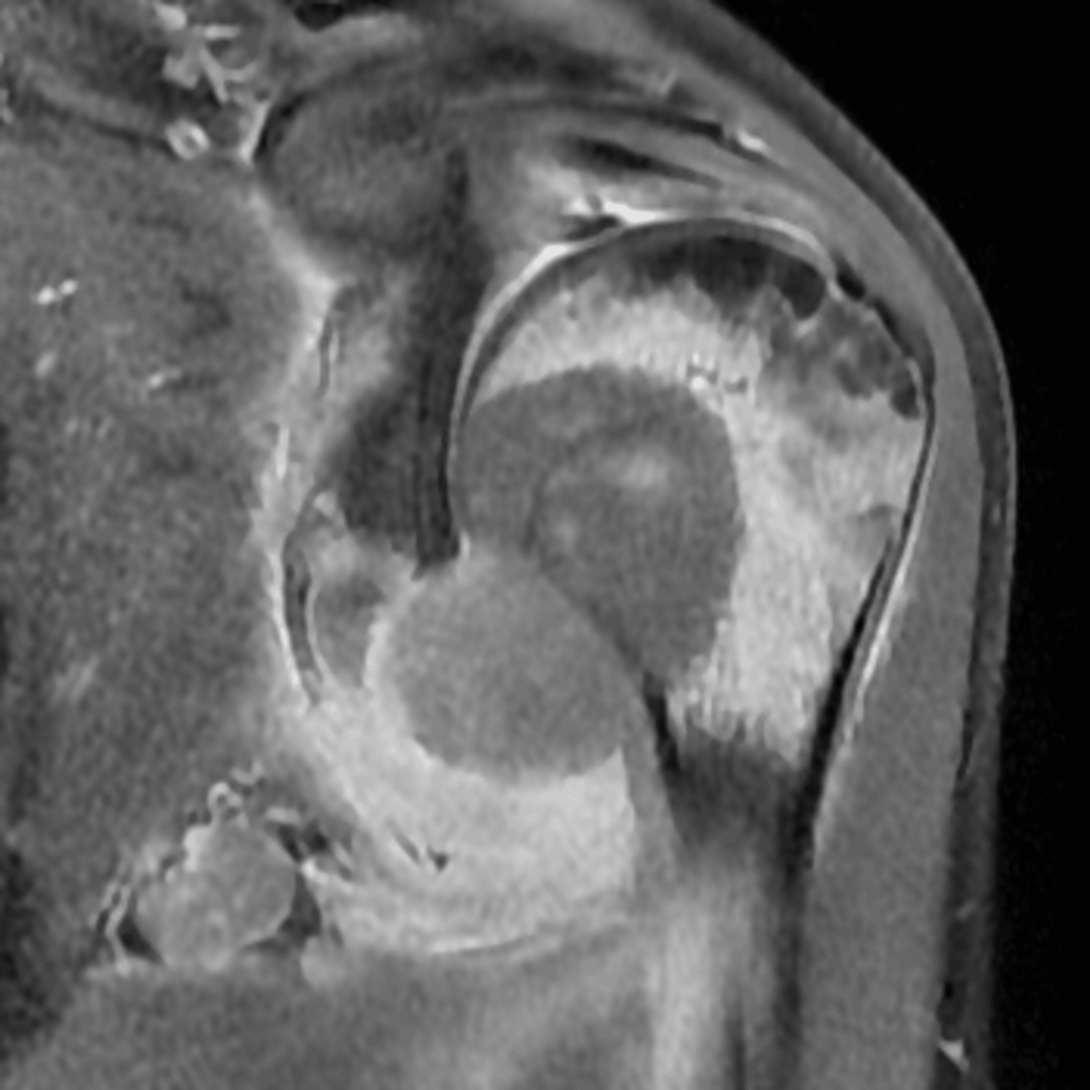
Post-contrast coronal T1-weighted fat saturated coronal image Mild uniform post-contrast enhancement of the mass is demonstrated.

**Figure 7:**
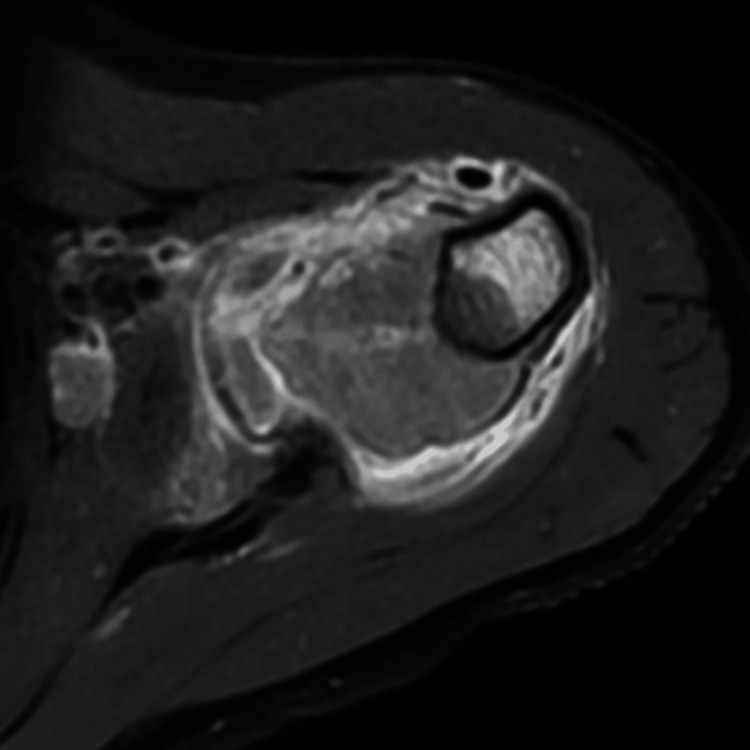
Post-contrast T1-weighted fat-saturated axial image of left shoulder Mildly enhancing mass and enlarged enhancing left axillary lymph node is seen.

MRI of the shoulder with contrast was performed using a 1.5-tesla MRI scanner (GE Medical System, 1.5T Signa Voyager GE Healthcare, Chicago, Illinois, USA). The study revealed a mass in the head and neck of humerus extending across the physeal scar with areas of erosion of the cortex (Figures 2, 3). Marked adjacent marrow edema was noted (Figure 4). The mass was seen extending into the subperiosteal space with resultant periosteal elevation. Few areas of breach of periosteum were also noted, suggesting invasive mass. The mass revealed restricted diffusion (Figure 5). On post-contrast scan, the mass displayed mild uniform contrast enhancement (Figure 6). Also noted was moderate glenohumeral joint effusion. Few enlarged lymph nodes were seen in the right axilla (Figure 7). Biopsy of the humeral mass revealed undifferentiated carcinoma.

**Figure 8:**
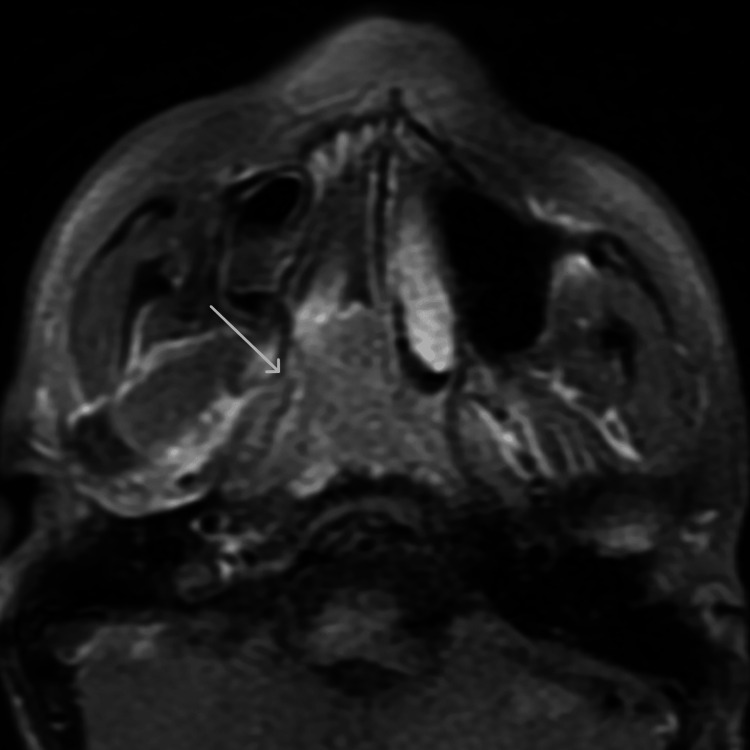
Post-contrast fat-saturated T1-weighted axial image through the skull base A heterogeneously enhancing invasive mass in the roof of nasopharynx with invasion of the right parapharyngeal space and right prestyloid recess is seen as pointed by the arrow.

**Figure 9:**
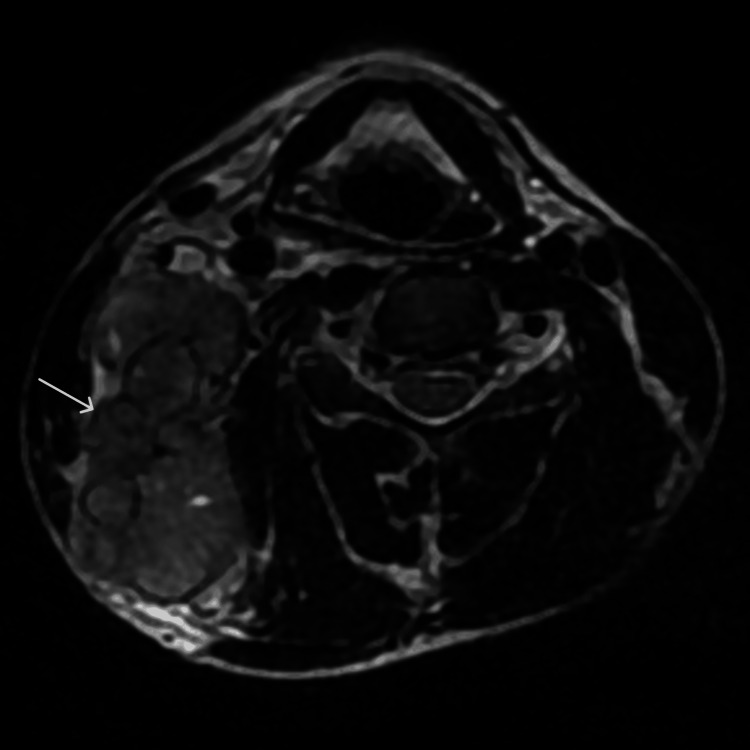
T2-weighted axial images through the neck A conglomerate of multiple enlarged lymph nodes is seen in the left deep cervical region (white arrow).

Subsequently, he had recurrent episodes of sneezing and running nose with occasional fever. A growing neck mass on the right was reported a month following onset of shoulder pain. MRI of the brain was performed for evaluation of the fainting episode. The study revealed heterogeneously enhancing soft tissue mass along the roof of nasopharynx invading the right parapharyngeal space and prestyloid space (Figure 8). The mass was seen encroaching upon the right posterior nares anteriorly. Enlarged multiple retropharyngeal lymph nodes were seen. A large lymph node mass was seen in the right deep cervical region (Figure 9).

The patient was referred to the oncology department, and complete assessment of extent of disease was done. Biopsy of the nasopharyngeal mass confirmed undifferentiated NPC. He also tested positive for EBV PCR, EBV VCA IgG and Epstein Barr nuclear antigen (EBNA) IgG. The case was discussed in tumor board, and T3 N3 M1a stage IVa was assigned. Six cycles of chemotherapy with Cisplatin and Gemcitabine were planned with probable future consideration for radiotherapy. He received chemotherapy with symptomatic improvement in shoulder movement and reduced pain.

## Discussion

NPCs usually present with neck mass, typically accompanied by symptoms such as nasal block, epistaxis, conductive hearing loss due to blockage of the eustachian tube and resultant middle ear effusion and cranial nerve palsies [8]. Presentation with humeral metastases is rare. The case underlines the importance of identifying unusual presentations of NPC and maintaining a high level of suspicion, especially in endemic areas including Southern China, Southeast Asia, North Africa and the Arctic [9].

Similar presentation of NPC was reported by Alherabi et al, the patient being a 23-year-old male presenting with a large shoulder mass that turned out to be metastatic NPC [10]. X-ray revealed an aggressive humeral lesion with pathological fracture [10]. Irawan et al. reported a similar case of a 33-year-old female presenting with a rapidly growing right shoulder mass [11]. The patient also complained of unilateral hearing impairment [11]. Our case differs from these studies as our patient belongs to pediatric age group and presented with shoulder pain without apparent mass. In our case, the initial presentation was confounding adding to delay in offering MRI imaging and hence delaying the proper diagnosis.

The common site for distant metastasis of NPC is to the bone, ranging from 54% to 80%. The most frequent metastatic sites were the rib (53.5%), thoracic vertebrae (50.0%), lumbar vertebrae (43.6%), pelvic girdle (42.3%) and lower limb (32.4%) [12]. Sham et al. reviewed records of 153 patients with NPC having skeletal metastases and concluded that younger patients have higher incidence of skeletal metastasis [13]. They reported overall 5% incidence of humeral metastasis of NPC including early and late presentations [12,13].

Synchronous and metachronous metastases have been described with NPC. Synchronous metastases are present at the time of initial diagnosis, whereas metachronous metastasis occur following definitive treatment after significant time interval [14]. The survival outcome is significantly influenced by both the number and location of the metastatic lesions, patients with metachronous metastasis having worse outcome as compared to synchronous [14].

Survival outcomes in these patients are often poor [10]. NPCs are radio-sensitive tumors. The mainstay of treatment for stage I NPC is external beam radiotherapy with additional chemotherapy in select cases, whereas cisplatin-based concurrent chemoradiotherapy is standard of care for locoregionally advanced disease [15]. Surgery has a little role in management of NPC other than for purposes of diagnostic biopsy, in radiation-resistant tumors and local recurrence.

## Conclusions

Humeral metastasis as a primary presentation of NPC is quite rare. Often, primary bone tumors are considered in children with an aggressive bone tumor in the humerus. Metastatic lesion of NPC in the proximal humerus can be a misleading initial presentation and may delay definitive management. A high index of suspicion is necessary regarding the awareness of the prevalence of NPCs in this age group and regarding the unusual presentation, especially in endemic regions. NPCs tend to be aggressive with a poor prognosis, and hence, it is crucial to avoid unnecessary delays in the diagnosis and treatment of NPC by being aware of the uncommon presentations.
